# A Children's Sermon. — Fairy Rings and Ringworms

**Published:** 1887-08-06

**Authors:** 


### THE DOCTOR'S PULPIT: A CHILDREN'S SERMON.

### FAIRY RINGS AND RINGWORMS.

The fairies are all gone out like the Dodo, but their rings remain. Can it be possible that any reader of The Hospital, great or small, does not know what a fairy ring is? It would seem a foolish question to ask, but there are so many people in London, even among the more comfortable classes, who know little or nothing about the country, that it may not be quite so unnecessary as it seems. It is not very long ago that a small cockney who saw a cow milked for the first time was disgusted to think that country people got their milk from a "nasty, dirty cow." He knew better, and took care always to have his from a "nice clean shop." A fairy ring (country folk forgive us!) is a ring of darker grass than that which is outside and inside it, and can generally be seen in old pastures: that is, in fields which have long been "laid down" in grass. Almost all little people in the country, and many big ones, when they see such a ring, immediately picture to themselves thousands of little fairy figures, with little fairy feet, tripping it airily and fairily at the midnight hour on the dewy grass. Round and round, and ever round they spin to the music of the flower-bells, that tinkle loudly enough for fairy ears to hear, though nobody else can catch the faintest sound. Just before the dawn of day Puck gives the order, and all the airy dancers vanish in a moment. But there is left behind, as a most convincing proof of their presence and their play, the fairy ring, with which the tiniest country child is as familiar as the town child is with her doll or her picture-book.

"And what can such a pretty ring as that left by the dancing of fairy feet have to do with such a nasty, filthy, tiresome thing as ringworm, which drives mother to distraction, and which the doctor seems as if he never would cure?" That is a little question, but might properly enough have a very large answer. The ringworm, or the little fungus that produces it, is not a "nasty, dirty, tiresome thing" at all, if it would only choose a different place to grow in. And it is just as near akin to the fairy ring as two Englishmen of the same trade, who carry on their business in different workshops. The little growth which produces the fairy ring is a fungus, and so is the small plant which produces the ringworm; and so is the puff-ball, and so too the mushroom, which everybody is so fond of. But surely nobody would wish us to eat the little things which produce the ringworm? If they could be collected in sufficient quantity, and stewed with steak like mushrooms, they might be just as nice to eat as the mushroom, for anything you know. Of course they might not, because some fungi (we prefer the word funguses, but that does not look learned, and some of the readers of The Hospital are dreadfully learned)—some fungi are safe to eat, and some are very poisonous.

What a curious thing it is that a child's head should be so much like a grass field, and that each of them may have such a peculiar ring in it! It is something for both big and little children to know that neither of these rings is produced by a *worm*, but by a plant—a cryptogamous plant. What in the world does cryptogamous mean? It means "without flowers." The little plant of the fairy ring and his little cousin of the ringworm never show any flowers. But they have plenty of "spores"; and everybody knows that a sound, healthy spore, if placed in right soil, will become a plant much sooner than a child will become a man. That is how the "ringworm" happens to be a "ring" at all. It is not a ring at first, but a little patch. The plant soon finishes all the nutrition it can find on the patch, and then begins to spread itself outwards, the central portion dying because it has exhausted the nutrition. So when the central little bit is dead there is nothing left but the ring round it, which constantly gets bigger and bigger unless a stop is put to it—that is, unless it is cured.

Now how is it to be cured? Whose fault is it that it is not cured? "Why, the doctor's, of course!" Softly, softly. It is often by no means the doctor's fault, nor the mother's, nor the nurse's, but the patient's. That is where the fault lies. Suppose we were going to try to stop the fairy ring from growing any bigger, what must we do? We must do one of two things: either dig up every bit of soil containing the little fungus which causes the ring, or else we must pour something into the ground strong enough to kill every little parent fungus, and every little baby fungus or spore. But you can see that if the ground had any feeling, which we may hope it has not, both these methods might be, and one of them certainly would be, very painful. Well, ringworm can only be cured in one of these two ways. Either we must "dig up" all the skin in which the fungus is growing, or we must put something into the skin which will be strong enough to destroy it. No doctor is such a monster as ever to propose to "dig up" the skin. So all of them do their best to put something into it to kill the fungus. And that is where the shoe pinches. The stuff which is going to cure the ringworm must be put *into* the skin. It is of no use whatever to pour it *on* the skin. Then if it must be put in, how is it to be done? We must begin by pulling out the hairs. That is not at all painful, because they are already loose. Then of course little holes are made where the hairs have come out, and if our stuff were strong enough, and would just run down of itself to the bottom of all these little holes, the thing might easily be done. But it won't; and so nothing more need be said on that point. It has to be "forced" down, and that is where all the pain and difficulty of curing ringworm really lie. That is how it is that the child is really to blame, and not the doctor or the mother. The doctor orders the stuff which is to effect the cure: and in nine cases out of ten it would be quite sufficient, if the patient would only submit to have it thoroughly used.

Of course it is painful, sometimes very painful, to have a smarting ointment or liquid brushed hard into your head with a stiff-bristled tooth-brush every day. And equally of course, the more frequently you brush the more painful it becomes, until the part is quite inflamed. Even so it must be. There are so many cures for ringworm, that if we were to name them all we might fill the page. But none of them, not one, is of any use unless it be thoroughly rubbed in. Because, of course, every separate fungus and every separate spore must be completely destroyed, otherwise the ringworm will all break out again; and these fungi and their spores grow right down in the little holes (follicles) from which the hairs spring. The stuff, therefore, must be forced in: and it must be either strong enough to kill the plant of itself, or it must be irritating enough to set up sharp inflammation, and the inflammation will swell up the parts in which the fungi and spores are growing, and so kill them. That is why this little sermon is addressed to children. They are the persons to be asked whether the ringworm is to be cured or not. The doctor will order the right stuff. Will you permit it to be rightly used? If you are true and heroic little Britons, you will: if you are poor little cowards, who cannot bear a twinge of pain, you will not; and then you will well deserve to keep your ringworm for two or three years.

(*To be continued.*)

